# FoMO and cyberstalking in adults: the mediating roles of early maladaptive schemas and phubbing

**DOI:** 10.3389/fpsyg.2026.1771852

**Published:** 2026-06-08

**Authors:** Lokman Koçak

**Affiliations:** Faculty of Education, Department of Educational Sciences, Bayburt University, Bayburt, Türkiye

**Keywords:** cyberstalking, early maladaptive schemas, FoMO, mediation, phubbing

## Abstract

**Introduction:**

It is noticeable that cyberstalking behavior, which has become widespread in recent years and has many negative effects, has been investigated to a limited extent. This study aimed to analyse the mediating role of early maladaptive schemas of abandonment and insufficient self-control and phubbing in the relationship between FoMO and cyberstalking among adult individuals.

**Methods:**

The study group comprised 540 adults aged 18–48 (71.1% female and 28.9% male). We adopted Pearson correlation analysis and PROCESS Macro Model 80 in regression analysis to explore the relationships among cyberstalking, FoMO, early maladaptive schemas and phubbing.

**Results:**

The results showed that there were significant relationships between the study variables. Also, results show that early maladaptive schemas of abandonment and insufficient self-control and phubbing mediate the relationship between FoMO and cyberstalking.

**Discussion:**

These findings suggest that the abandonment schema may be associated with FoMO-related cyberstalking through a theoretically informed pathway that may reflect attachment-related vulnerabilities, whereas the insufficient self-control schema may reflect a possible self-regulation-related dimension that may be associated with FoMO-related digital behaviors of this process. The inclusion of phubbing in the model further indicates that FoMO-related attentional and relational dysregulation may be linked to monitoring-oriented online behaviors. In this regard, these findings may provide a theoretical perspective for future studies examining attachment-related schema sensitivities, self-regulation processes, and mindful technology use in relation to FoMO-related digital behaviors.

## 1 Introduction

The use of social media has spread rapidly in recent years and has become an indispensable part of daily life. Despite the many risks in terms of privacy, exponential growth and security (Silva Santos et al., 2023), it is an inevitable fact that the demand for social media platforms is increasing day by day. Recent global statistics indicate that internet and social media use have reached unprecedented levels worldwide. According to Statista (2025), approximately 5.52 billion individuals-out of a global population of 8.08 billion-use the internet, and about 63.8% of the world’s population are active social media users. Among social media platforms, Facebook remains the largest with approximately 3.6 billion users, followed by YouTube (2.5 billion), Instagram (2 billion), and TikTok (1.6 billion). Similar trends are also observed in Türkiye. Current statistics indicate that the most widely used social media platforms in Türkiye are YouTube (57.5 million users), Instagram (57.1 million), TikTok (37.7 million), Facebook (34 million), and X (20 million).

Social media tools, which can be used for many purposes such as initiating and maintaining communication, conducting research, planning social activities, and entertainment (Başoğlu and Yanar, 2017), also provide individuals with opportunities to initiate romantic relationships or maintain existing relationships in online environments (Spitzberg and Cupach, 2007; Smoker and March, 2017). Features such as location sharing and tagging on social media platforms enable users to access information about others’ activities and whereabouts, thereby facilitating repetitive and monitoring-oriented observation of other individuals’ behaviors in digital environments. Similar to traditional stalking, stalking behaviors in online environments may include actions such as controlling, manipulating, coercing, persistently observing, and repeatedly attempting to contact another person (Smoker and March, 2017; March et al., 2020). These behaviors are commonly referred to as cyberstalking in the literature. Cyberstalking is generally defined as the repeated use of electronic communication technologies to monitor, harass, threaten, or otherwise intrude upon another individual (Dreßing et al., 2014; Duffy et al., 2023). In the present study, cyberstalking is conceptualized as repetitive and monitoring-oriented online behavior involving intrusive digital surveillance, excessive tracking of others’ online activities, and boundary-crossing interpersonal monitoring. Although the literature includes specific subtypes such as intimate partner cyberstalking, the current study addresses cyberstalking as a broader pattern of intrusive online monitoring behavior rather than restricting it to romantic relationship contexts.

When reviewing the literature on cyberstalking, it is evident that studies have developed around different sub-trends. Among the subtypes discussed in the literature, studies focusing on intimate partner cyberstalking, intrusive online surveillance behaviors, and repetitive patterns of boundary-crossing digital monitoring and harassment are particularly prominent (Spitzberg and Cupach, 2007; Smoker and March, 2017). A significant portion of these studies address cyberstalking in the context of interpersonal control, accessibility, and boundary violation.

When the studies on cyberstalking are examined, it is seen that gender variable is mostly addressed and women tend to cyberstalking more than men (March et al., 2020; Smoker and March, 2017). In some studies, it was found that the frequency of stalking behaviors was equal between genders (Shorey et al., 2014). In studies conducted with personality traits, it has been determined that the dark quartet (Machiavellianism, narcissism, psychopathy and sadism) is an important predictor of cyberstalking (Smoker and March, 2017; March et al., 2020). In Silva Santos et al.'s (2023) study, it was found that there was a significant relationship between social media use and FoMO and cyberstalking and that these two variables predicted cyberstalking.

### 1.1 FoMO and cyberstalking

In addition to communication, social media also provides information about the daily activities of other individuals (Çakır, 2020). Individuals are curious about where others go, which leads to an increase in screen time. The increase in screen time has led current studies to focus on the concept of fear of missing out (FoMO) to determine whether technology is harmful for adults (Elhai et al., 2021). FoMO is defined as the concern about others’ rewards for being on social media and the desire to be in constant contact with others (Przybylski et al., 2013). In the context of FoMO, individuals fear losing likes and rewards on social media (Rozgonjuk et al., 2020).

FoMO causes various negative consequences, such as the negative impact of social media on daily life and productivity (Rozgonjuk et al., 2020). While empirical results to confirm this theory are not yet available, Alutaybi et al. (2020) point out that certain FoMO motivations (e.g., seeking popularity or having information) can lead to unhealthy behaviors such as cyberstalking. Thus, it can be hypothesized that one of the factors that may lead a person to cyberstalk is the fear of being excluded from important moments in the lives of those they are interested in. In this context, FoMO can be considered a motivational process that increases individuals’ need for interpersonal control and their expectation of being accessible, thereby paving the way for the continuation of online monitoring and tracking behaviors. In this context, it can be thought that FoMO may increase the likelihood of cyberstalking and may be associated with cyberstalking-related tendencies. Limited research on FoMO and cyberstalking also shows that there is a positive and significant relationship between the two variables (Bibi et al., 2023; Gómez-Yepes et al., 2025; Silva Santos et al., 2023).

### 1.2 The role of early maladaptive schemas

Schema theory suggests that there are universal basic needs that need to be met in the early years of life in order for the individual to lead a mentally healthy and harmonious life. These basic needs are expressed as secure attachment, autonomy, spontaneity and play, perception of competence and identity, freedom and realistic boundaries (Young and Klosko, 2011). As a result of basic needs not being met or being prevented in a traumatizing way, strong thought patterns/beliefs called schemas are formed. Negative experiences and maltreatment in early years are also effective in the formation of schemas (Young et al., 2003). It is stated that early maladaptive schemas, which are rigid, destructive and very difficult to change, are self-repetitive and survival-oriented patterns (Young and Klosko, 2011). Although painful for the individual, schemas provide a sense of consistency and control to the individual because they are comforting and familiar at the same time (Young et al., 2003). Although early maladaptive schemas seem to be adaptive in this respect, they have a faulty, dysfunctional and limiting structure that damages the individual’s self-perception and relationships with others (Farrell et al., 2015). According to the content areas, five schema domains and 18 early maladaptive schemas were identified (Young et al., 2003).

It is stated that early maladaptive schemas closely affect the individual’s emotions, thoughts, behaviors and the way of relating with the outside world (Young, 1999). In other words, it can be said that individuals evaluate life events and relationships according to their schemas. With the increase in the amount of time adults spend on social media, it has become important to consider these cognitive patterns in conjunction with the meanings attributed to online interactions and interpersonal experiences in digital environments. Schemas shaped by parental attitudes, the social environment in which individuals are raised, and hereditary characteristics provide a framework related to how individuals perceive and interpret interpersonal situations on social media platforms (Young and Klosko, 2011). Cyberstalking is defined as a repetitive pattern of digital interaction in which interpersonal intimacy, accessibility, and boundaries of control are continuously violated, rather than being an impulsive, momentary behavior (Spitzberg and Cupach, 2007; Storey et al., 2009). Approaches that focus solely on impulsivity or general personality traits may be limited in understanding such persistent and boundary-crossing digital behaviors. Early maladaptive schemas, on the other hand, provide a more enduring cognitive framework that encompasses an individual’s interpersonal threat perceptions and sensitivities related to abandonment or loss of control, thus providing a distinctive context for conceptualizing the psychological patterns associated with cyberstalking (Farrell et al., 2015; Young et al., 2003). However, despite their conceptual relevance, schema-based explanations have rarely been examined within a structured serial pathway that integrates FoMO-related motivational processes and interpersonal digital disengagement patterns in explaining cyberstalking behaviors.

### 1.3 The role of phubbing

FoMO leads to negative emotions such as social exclusion, incompleteness, and social comparison, which may increase the individual’s behavior of controlling others’ activities on online platforms (Przybylski et al., 2013). In this context, individuals may develop cyberstalking behavior by intensively following the lives of others on social media. However, it is thought that this relationship may have not only cognitive but also behavioral mediators. At this point, phubbing behavior draws attention. Phubbing is defined as paying attention to the phone in the presence of two or more people and communicating with the phone rather than the people in the environment (Blanca and Bendayan, 2018). Roberts and David (2022), on the other hand, explain phubbing as an individual’s paying attention to his/her mobile device during face-to-face social interaction and putting social relationships in the background. Hızarcı (2018) stated that individuals with problematic mobile phone use have anxious attachment and fear of missing developments. Many studies show that phubbing behavior is associated with FoMO (Albalá-Genol et al., 2025; Batmaz et al., 2024; Blanca and Bendayan, 2018; González et al., 2025; Tandon et al., 2022; van der Schyff et al., 2022) and causes the individual to focus more on the online social world. As individuals with high FoMO level do not get enough satisfaction from face-to-face social relationships, they tend to control the lives of others through mobile devices, which may increase the risk of cyberstalking. Therefore, phubbing may function as a mediating mechanism that transforms an individual’s FoMO-induced social dissatisfaction into digital control behaviors. In this context, phubbing behavior is conceptualized not only as social avoidance or a shift toward technology, but also as difficulty regulating attention and a shift in interaction from interpersonal contexts to digital environments. This shift in attention may increase feelings of neglect and insecurity in face-to-face relationships, potentially paving the way for more intense monitoring and control-focused behaviors in online environments. This interpersonal neglect experience may represent a theoretical pathway through which phubbing-related interpersonal disengagement could be associated with monitoring-oriented cyberstalking behaviors, particularly when considered together with early maladaptive schemas such as abandonment and insufficient self-control.

Repeated phubbing behavior may gradually shift individuals’ attention away from face-to-face interaction toward digitally mediated environments. Over time, this attentional shift may weaken the quality of direct interpersonal engagement and increase individuals’ reliance on online contexts for monitoring social information. In such situations, individuals may increasingly turn to repetitive and intrusive monitoring of others’ activities in digital environments in order to maintain a sense of connection, awareness, or control. This tendency may be associated with monitoring-oriented online behaviors, including cyberstalking.

Phubbing behavior is thus considered not only as a problematic form of technology use, but also as an interpersonal situation related to experiences of perceived neglect and being sidelined in face-to-face interactions (Chotpitayasunondh and Douglas, 2018; Roberts and David, 2022). In this context, FoMO acts as a motivational process that increases individuals’ need for interpersonal control and their expectation of being accessible, and may be associated with the continuation of online monitoring and tracking behaviors. It is thought that such experiences may be related to how individuals evaluate their social interactions in online environments and may contribute to understanding interpersonal sensitivities in the digital context. In this context, considering phubbing behavior alongside early maladaptive schemas such as abandonment and insufficient self-control may provide a conceptual framework for examining variables related to cyberstalking together.

## 2 Present study

It has been reported that individuals exposed to cyberstalking may experience depression, anxiety, sleep and eating disorders, as well as various negative psychological, social, and physical consequences (March et al., 2020). Furthermore, findings indicate that victims of cyberstalking are more likely to experience post-traumatic stress symptoms (Short et al., 2015). It is noted that these individuals generally report lower life satisfaction and psychological resilience levels; this situation can be considered in conjunction with a cyclical pattern of emotional distress that increases sensitivity to negative social interactions (Olson et al., 2024; van Baak and Hayes, 2018). These findings highlight the importance of examining the psychological processes associated with cyberstalking in greater detail.

With the integration of digital technologies and social media into the center of daily life, it is emphasized that the behavioral patterns individuals develop in online environments are related to various psychological processes. In this context, Fear of Missing Out (FoMO) is considered an experience associated with individuals showing a more intense tendency toward monitoring-oriented online behaviors and excessive following of others’ activities due to the fear of missing out on social experiences (Przybylski et al., 2013). Although the relationship between FoMO and cyberstalking has been addressed in a limited number of studies (Bibi et al., 2023; Gómez-Yepes et al., 2025; Silva Santos et al., 2023), current findings indicate that these two variables can be evaluated together. However, it is thought that explaining the relationship between FoMO and cyberstalking solely through situational or cognitive processes may not be sufficient; cognitive structures based on the individual’s developmental history and behavioral patterns accompanying technology use should also be considered.

In this context, early maladaptive schemas provide an important cognitive framework for understanding how individuals interpret experiences related to FoMO. Two schema domains are particularly noteworthy in this regard. The abandonment schema encompasses the individual’s core beliefs that they will be abandoned by important people or left emotionally alone (Young et al., 2003; Bach et al., 2018). It is noted that this schema is associated with intense attachment anxiety and sensitivity to rejection in interpersonal relationships. It is stated that individuals with high FoMO levels may show a tendency to follow the lives of others more closely in online environments due to anxiety about social exclusion or being forgotten; this tendency may be more pronounced among individuals with a strong abandonment schema.

The insufficient self-control/self-discipline schema is defined as a cognitive pattern in which the individual struggles to regulate their impulses, pleasure-seeking tendencies, and attention (Young et al., 2003; Bach et al., 2018). It is noted that this schema may be associated with problematic technology use when considered alongside uncontrolled behavior patterns in highly stimulating digital environments (Wegmann et al., 2017). In individuals with high levels of FoMO, this schema may represent a cognitive vulnerability associated with the persistence of online monitoring and tracking behaviors.

In addition to these cognitive patterns, phubbing behavior also emerges as a noteworthy variable in the context of FoMO and cyberstalking. Phubbing is defined as an individual directing their attention to their mobile device during face-to-face social interaction and placing interpersonal interaction in the background (Blanca and Bendayan, 2018; Roberts and David, 2022). The literature has shown that phubbing behavior is associated with loneliness, low relationship quality, and FoMO (Alt and Boniel-Nissim, 2018; Chotpitayasunondh and Douglas, 2018). These findings suggest that phubbing behavior can be considered not only as a problematic form of technology use but also within a context that can be examined alongside experiences of perceived neglect and being sidelined in interpersonal interactions. In this context, it can be argued that phubbing behavior, when evaluated alongside early maladaptive schemas, may provide a conceptual framework for how variables associated with cyberstalking can be addressed together.

In the literature related to cyberstalking, various risk factors such as impulsivity, general attachment insecurity, dark personality traits, and problematic social media use (Brodie and Scott, 2024; Duffy et al., 2023; Kircaburun et al., 2018) are frequently emphasized. However, these variables are mostly addressed within the framework of individual tendencies or personality-based predispositions, offering limited explanation of how cyberstalking is understood in an interpersonal context. Early maladaptive schemas, as developmentally rooted cognitive frameworks associated with interpersonal threat perceptions and emotional regulation processes, may provide a theoretical context for understanding certain digital behaviors within a broader psychological framework (Bach et al., 2018; Farrell et al., 2015; Pilkington et al., 2020; Young et al., 2003). Considering this cognitive framework alongside interpersonal neglect and background experiences such as phubbing allows for the examination of variables related to cyberstalking not only in terms of individual predispositions but also within the context of interpersonal interpretation processes.

While previous studies have examined FoMO, problematic technology use, and specific personality-based vulnerabilities in the context of cyberstalking, these variables have largely been addressed separately. The present study does not seek to introduce entirely new predictors; rather, it examines developmentally rooted cognitive schemas (abandonment and insufficient self-control) together with phubbing, conceptualized as a digitally situated behavioral pattern with interpersonal implications, within a unified model. Within this framework, the study adopts a more holistic perspective by evaluating cyberstalking not only in terms of trait-based tendencies but also in relation to attachment- and self-regulation–based cognitive processes. To our knowledge, studies testing these variables together within a structured serial framework remain limited.

The study aims to examine the mediating effect of early maladaptive schemas and phubbing in the relationship between FoMO and cyberstalking. In this direction, abandonment and insufficient self-control schemas and phubbing will be discussed in the study. The abandonment and insufficient self-control schemas were specifically selected because they appear conceptually relevant to the psychological processes underlying FoMO and monitoring-oriented online behaviors. The abandonment schema reflects fears related to rejection, exclusion, and emotional disconnection, which conceptually overlap with the social anxiety and interpersonal sensitivity dimensions frequently associated with FoMO. In contrast, the insufficient self-control schema is associated with difficulties in impulse regulation, attentional control, and behavioral restraint, which may be relevant to compulsive monitoring and excessive engagement in digital environments. Therefore, these two schemas were considered theoretically more compatible with the attachment-related and self-regulatory dimensions of FoMO-related cyberstalking tendencies. Previous studies reporting associations between these schemas and problematic smartphone use, smartphone addiction, problematic Facebook use, and problematic internet use were considered supportive rather than determinative in the selection of these schemas (Arpacı, 2021, 2023; Cudo et al., 2020; Shajari et al., 2016); (Arpacı, 2023), smartphone addiction (Arpacı, 2021) and problematic facebook use (Cudo et al., 2020). Accordingly, this study aims to examine the mediating role of early maladaptive schemas and phubbing in the relationship between FoMO and cyberstalking in adults. In this context, the following hypotheses were proposed:

*H1*: FoMO directly effects cyberstalking (i)

*H2*: FoMO indirectly effects cyberstalking through abandonment (a-b)

*H3*: FoMO indirectly effects cyberstalking through insufficient self-control (e-f)

*H4*: FoMO indirectly effects cyberstalking through phubbing (c-d)

*H5*: FoMO indirectly effects cyberstalking through abandonment and phubbing (a-g-d)

*H6*: FoMO indirectly effects cyberstalking through insufficient self-control and phubbing (e-h-d)

## 3 Materials and methods

### 3.1 Study design

The study was conducted using a cross-sectional design within the framework of quantitative research methods. Therefore, the proposed serial mediation model should be interpreted in terms of statistical associations among variables rather than causal or temporal relationships. Criteria such as being 18 years of age or older, using the internet every day and having at least one social media account were taken into consideration. These inclusion criteria were established to ensure that participants had regular access to and experience with the relevant online contexts, given that the variables examined in the study (FoMO, phubbing, and cyberstalking) are directly related to digital environments and social media interactions.

### 3.2 Participants

The study group consisted of a total of 540 adults, 384 female (71.1%) and 156 male (28.9%) between the ages of 18 and 48 (M = 27.35, SD = 7.45), and of the participants, 155 (28.7%) were married and 385 (71.3%) were single. The sample represents a mix of socioeconomic backgrounds from the youngest to the oldest age. The sample was highly ethnically homogeneous and consisted entirely of Turkish adults.

### 3.3 Measures

#### 3.3.1 Demographic information form

Participants filled in demographic information such as gender, age, marital status, daily internet usage time, most frequently used social media platform.

#### 3.3.2 Cyberstalking scale

The scale developed by Silva Santos et al. (2023) was adapted to Turkish culture by Koçak et al. (2023). The trial form of the scale, which was developed for adult individuals, consists of 15 items and is 5-point Likert type. The final version of the scale consists of 10 one-dimensional items. When the EFA results were examined, it was determined that the factor loadings ranged between 0.44 and 0.88 and the total variance explained by the one-factor scale was 37.65%. These results indicate that the scale shows acceptable level features in the context of EFA. When the CFA results are examined, it is seen that *χ*^2^ = 52.415, df = 32, CFI = 0.95, IFI = 0.95 and GFI = 0.93 values show excellent fit; RMSEA = 0.068, RMR = 0.080, SRMR = 0.056, NNFI = 0.93 and AGFI = 0.89 values have acceptable fit level (Gürbüz, 2019; Seçer, 2015). In the reliability analyses, the internal consistency coefficients for EFA and CFA were determined as 0.81 and 0.83, and the two-half test reliability coefficients were determined as 0.77 and 0.76, respectively.

#### 3.3.3 Fear of missing out scale (FoMO) scale

FoMO scale Przybylski et al. (2013) was developed by. The internal consistency of the developed scale is *α* = 0.90. The validity and reliability study of the Turkish version of the scale was conducted by Gokler et al. (2016). The one-dimensional scale developed consists of 10 questions and its items are graded from 1= “not at all true” to 5 = “extremely true.” The scores of the participants from the scale vary between 10 and 50 and there is no cut-off score. An increase in the score obtained from the scale means that the probability of the individual’s fear of missing out increases. The factor loads of the SSCS range from 0.36 to 0.77, Cronbach’s alpha coefficient = 0.81, test–retest reliability coefficient = 0.8. The analyzes showed that the FoMO is a valid and reliable measurement tool for university students (Gokler et al., 2016). In this study, Mc. Donald *ω* and Cronbach’s Alpha values were calculated as 0.82.

#### 3.3.4 Generic scale of phubbing

The Generic Scale of Phubbing was developed by Chotpitayasunondh and Douglas (2018) and adapted to Turkish culture by Ergün et al. (2019). The GSP consists of a 15-item scale comprising four subscales: phubbing, nomophobia, interpersonal conflict, self-isolation, and acceptance of problems. Participants rated each item on a 7-point Likert-type scale ranging from 1 (never) to 7 (always), with higher scores indicating greater levels of phubbing. Construct validity studies on the Turkish adaptation of the scale showed that the factor structure of the scale was generally preserved and that the fit indices were at an acceptable level (χ^2^/df = 1.99–2.04, RMSEA = 0.07–0.08, SRMR = 0.06–0.07, CFI = 0.95–0.96), and internal consistency Cronbach’s α values ranged from 0.78 to 0.87, supporting the psychometric adequacy of the scale. The adaptation study found satisfactory Cronbach’s alpha coefficients for the subscales: Nomophobia (0.82), interpersonal conflict (0.87), self-isolation (0.81), and awareness of problems (0.70). A higher score on the scale indicates a higher level of negative behaviors. In the present study, the total phubbing score was used in the analyses in order to evaluate overall phubbing tendencies rather than specific subdimensions and to maintain a more parsimonious mediation model structure.

#### 3.3.5 Young schema scale short version-3

Young schema scale was developed by Young and Brown (2005). Turkish adaptation studies were conducted by Soygüt et al. (2009) with 1,071 university students between the ages of 17–35, 597 of whom were female, 469 of whom were male, and 5 of whom did not specify gender. Items on the schema scale used to assess early maladaptive schemas were answered using a 6-point Likert-type rating scale. Participants were asked to rate each statement on a scale ranging from 1= “Completely untrue for me” to 6= “Describes me perfectly.” High scores on the scale indicate that the cognitive patterns associated with the relevant schema are more pronounced. In the Turkish adaptation study, the construct validity of the scale was examined using Exploratory Factor Analysis (EFA). As a result of the Equamax-rotated principal component analysis, a 14-factor structure was obtained that largely corresponded to the theoretical structure of the original scale, and it was reported that these factors explained approximately 49.11% of the total variance. Furthermore, high-order factor analyses revealed that early maladaptive schemas clustered under five superordinate schema domains (disconnection and rejection, impaired autonomy and performance, impaired boundaries, other-directedness, and hypervigilance). The criterion (convergent) validity of the scale was assessed by examining the relationships between the YSQ-SF3 subscales and the SCL-90-R global symptom index and subscales. The findings showed positive and significant correlations between early maladaptive schemas and psychological symptom levels, consistent with theoretical expectations (*r* = 0.19–0.62, *p* < 0.01). These results indicate that the scale consistently reflects variables related to the psychological constructs it aims to measure. Within the scope of discriminant validity, the YSQ-SF3 scores of clinical and non-clinical samples were compared, and it was determined that the clinical group scored significantly higher on schemas such as abandonment, social isolation/insecurity, emotional deprivation, and poor self-control. This finding demonstrates the scale’s ability to distinguish between different psychological groups.

The reliability of the YSQ-SF3 was assessed using internal consistency and test–retest methods. The Cronbach’s alpha coefficients calculated for the subscales ranged from 0.63 to 0.80, and for the upper schema domains, they ranged from 0.53 to 0.81. In the test–retest application conducted at three-week intervals, meaningful correlation coefficients ranging from 0.66 to 0.82 were obtained for the subscales (*p* < 0.01). These findings indicate that the scale is a stable and reliable measurement tool over time. Based on this information, it was concluded that the scale was valid and reliable. In this study, only abandonment (5 items) and insufficient self-control (5 items) schemas were used. The items numbered 2, 20, 38, 56, and 73 measure the abandonment schema; items 15, 33, 51, 69, and 87 measure the insufficient self-control schema.

### 3.4 Procedure

The data were collected by the researcher through an online form. Convenient sampling method was used in the study. Participants were reached via an online survey link; the link was announced through social media platforms and online communication channels to encourage voluntary participation. Information such as instructions on how to answer the measurement tools, the purpose and content of the research were written on the first page of the form. It was also added on the first page of the form that participation in the study was voluntary and the results obtained from this study would be used in a scientific research. After the explanations, individuals who volunteered participated in the study. Before the analyses, the dataset was screened for incomplete or invalid responses. Forms containing substantial missing data or clearly inconsistent response patterns were excluded from the final dataset. In addition, repeated submissions were checked and controlled during the data screening process to reduce the possibility of duplicate responses in the online form. Participants were also informed that their responses would remain anonymous and confidential in order to encourage more accurate and unbiased self-reporting during data collection.

### 3.5 Statistical analysis

In determining whether the distribution is suitable for parametric tests, descriptive methods (Abbott, 2011), which include skewness, kurtosis, mean and standard deviation values, were used. The fact that the skewness values of the variables are between 0.01 and 1.10 and the kurtosis values are between −0.30 and 0.96 and these values are between −1.5 and +1.5 means that the data are at an acceptable level in terms of normality (Tabachnick and Fidell, 2020). As a result of the correlation analysis, it was determined that there was a positive and significant (*p* < 0.01) relationship between cyberstalking and FoMO (*r* = 0.36), abandonment (*r* = 0.35), insufficient self-control (*r* = 0.25) and phubbing (*r* = 0.37). The correlation values obtained reveal that a mediation model can be established between the variables considered in the study. Findings regarding normality assumptions are shown in Table 1.

**Table 1:** Descriptive statistics, reliability and correlations between study variables (*N* = 540).

Variable	1	2	3	4	5	*α*	M	SD	Skew	Kurt
1. CS	—	0.36**	0.35**	0.25**	0.37**	0.84	26.22	7.41	0.01	−0.30
2. F		—	0.41**	0.23**	0.51**	0.90	18.18	8.42	1.10	0.96
3. A			—	0.21**	0.46**	0.66	11.20	3.72	0.71	0.65
4. IS				—	0.39**	0.66	14.30	4.29	0.05	−0.26
5. P					—	0.90	40.54	14.83	0.56	−0.19

*p < 0.05, **p < 0.01. A, Abandonment; *α*, Cronbach’s Alpha; CS, cyberstalking; F, FoMO; IS, insufficient self-control; P, phubbing; SD, standard deviation.

Following the normality assumptions, the data set was reviewed for multicollinearity and the assumptions of regression analysis were examined. In line with the findings obtained, it was determined that Tolerance values were greater than 0.10 and VIF values were less than 10. Then, it was seen that the Durbin-Watson value was determined between 1 and 3, which is the criterion range, and thus the data set was suitable for regression analysis in this respect (Seçer, 2015). Within the scope of these results, it is concluded that the data set is normally distributed and there is no multicollinearity problem in the data set. In addition, the data were examined in terms of outliers, linearity, homoscedasticity, and residual independence before the main analyses. The findings indicated no serious violation of the assumptions required for the regression-based mediation analyses conducted in the study. The results regarding the assumptions of the regression analysis are given in Table 2.

**Table 2:** Results of regression assumption tests.

Model (DV: Cyberstalking)	Durbin–Watson
	1.72

Independent variable	Tolerance	VIF
FoMO	0.68	1.46
Abandonment	0.79	1.27
Insufficient self-control	0.89	1.12
Phubbing	0.67	1.50

**Figure 1:**
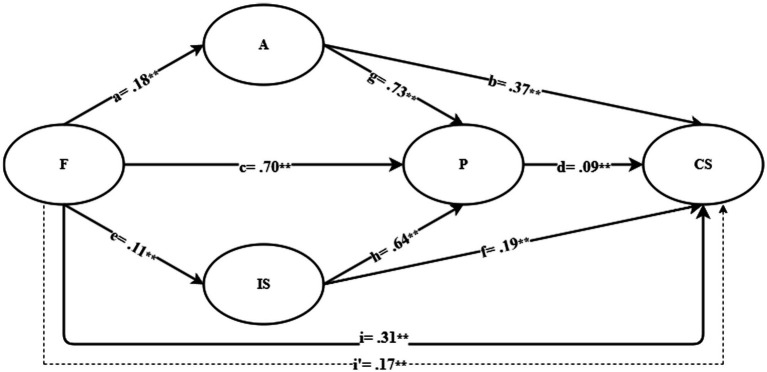
Multiple serial mediation model 80.

In the study, the mediation model of the factors predicting the cyberstalking level of adult individuals was analyzed with the Process macro software developed by Hayes (2018), which is an add-on of the SPSS program. In this analysis, the distribution of 5,000 randomly generated data drawn from the original data set with the Bootstrap (resampling) technique was tested at 95% confidence interval. In this model, Baron and Kenny's (1986) criteria were taken as reference. Accordingly, as a prerequisite for the mediation effect, the dependent variable and the independent variable should be significantly related to each other, the relationship between the mediator variable and the independent variable should be significant, and the dependent variable should be significantly related to both the independent variable and the mediator variable. In the study, regression analysis based on the bootstrap method was applied to describe the mediating role of early maladaptive schemas and phubbing in the relationship between FoMO and cyberstalking. This method was preferred because it estimates the confidence intervals of the indirect effect more reliably than the Sobel test and is less dependent on the assumption of normal distribution (Gürbüz, 2019). Model 80 in Process macro software was used in mediation analyses. The main reason for choosing Model 80 is that early maladaptive schemas (abandonment and insufficient self-control) are seen not only as mediating variables in the relationship between FoMO and cyberstalking but also as higher-level cognitive structures that underpin phubbing behavior. Therefore, it is believed that FoMO is linked to early maladaptive schemas; these schemas may increase phubbing behavior, and phubbing could also be connected to cyberstalking. Thus, Model 80 was considered more appropriate than simple parallel mediation models because it allows the simultaneous testing of both parallel (M1 and M2) and serial (M1/M2 → M3) indirect pathways within a single analytical framework (Figure 1).

The hypothesized model was tested using Hayes’ (2018) PROCESS macro for SPSS. PROCESS applies ordinary least squares regression to estimate model coefficients and to compute direct and indirect effects in mediation and moderation analyses. Model 80 enables the examination of a combined parallel and serial multiple mediation structure. In the present study, Model 80 was employed to test a parallel and serial mediation model (Figure 1). The model includes nine direct effects (paths a–i). Specifically, it incorporates two parallel mediators (M1 and M2), both of which are serially antecedent to a third mediator (M3). Several specific indirect effects are estimated within this framework. These indirect effects are calculated as the product of the relevant path coefficients along each mediational sequence. The specific indirect pathways examined between X and Y correspond to those reflected in the title of the present study. Accordingly, cyberstalking was determined as the dependent variable (Y), FoMO as the independent variable (X), and abandonment, insufficient self-control and phubbing as the mediating variable (M). In additional analyses, gender, age, marital status, daily internet use time, and most frequently used social media platform were included in the model as covariates to control for potential confounding effects. In addition, a *post-hoc* statistical power analysis was conducted based on the most complex outcome model in the study. Considering the observed effect size (*R^2^* = 0.22), four predictors, an alpha level of 0.05, and a sample size of 540, the analysis indicated that the study had sufficient statistical power to detect medium effect sizes within the proposed model. To evaluate the potential effect of common method bias, Harman’s single-factor test was conducted. The results indicated that the first unrotated factor accounted for 23.53% of the total variance, which is below the commonly suggested threshold of 50%. Therefore, common method variance was not considered a serious concern in the present study.

## 4 Results

Figure 1 contains the unstandardized regression coefficients for each path in the model. Together, the predictors in the model explain 22% of the variance in cyberstalking. Overall, the findings suggest that the relationship between FoMO and cyberstalking can be explained both directly and indirectly through early maladaptive schemas and phubbing. When the findings are examined individually, it is seen that FoMO is significantly and positively associated with cyberstalking (*β* = 0.31, SE = 0.04, *p* < 0.001). In this case, H1 (i) was confirmed. The relationship between FoMO and cyberstalking has been found to be indirectly significant through abandonment schema (*β* = 0.06, SE = 0.02, *p* < 0.001), insufficient self-control schema (*β* = 0.02, SE = 0.01, *p* < 0.001) and phubbing (*β* = 0.06, SE = 0.01, *p* < 0.001).

When the indirect effects of the mediating variables were examined at the 95% confidence interval, the bootstrap confidence intervals for abandonment (95% CI = 0.03, 0.10), insufficient self-control (95% CI = 0.00, 0.04), and phubbing (95% CI = 0.03, 0.10) did not include zero. Therefore, the specific indirect effects were considered statistically significant in the model. In this case, H2 (a-b), H3 (e-f), and H4 (c-d) were confirmed. Furthermore, the relationship between FoMO and cyberstalking was found to be significant through serial indirect paths via abandonment schema and phubbing (*β* = 0.01, SE = 0.00, *p* < 0.001) and insufficient self-control schema and phubbing (*β* = 0.01, SE = 0.00, *p* < 0.001). In this context, when the indirect effects of the mediating variables were examined at a 95% confidence interval, it was determined that the mediating effects were significant in the model, since there were no zero values between the abandonment schema and phubbing (95%CI = 0.00, 0.02) and the insufficient self-control schema and phubbing (95%CI = 0.00, 0.01) were found to be non-zero, confirming that serial indirect effects were significant in the model. Accordingly, both pathways within the series of indirect effects—FoMO → abandonment schema → phubbing → cyberstalking and FoMO → insufficient self-control schema → phubbing → cyberstalking—are significant (Table 3). In this case, H5 (a-g-d) and H6 (e-h-d) are confirmed. When specific indirect effects are evaluated together, it is seen that the indirect paths calculated through the abandonment schema and phubbing are relatively more pronounced, while the indirect effects obtained through the insufficient self-control schema remain smaller in magnitude. Considering the effect sizes in According to the effect sizes presented in Table 3, the indirect effect observed in the FoMO → abandonment schema → cyberstalking pathway (effect = 0.07) was relatively higher than the other specific indirect effects examined in the study, followed by the FoMO → phubbing → cyberstalking pathway (effect = 0.06). The serial indirect effects (effect = 0.01) were statistically significant; however, their magnitudes were relatively small and should therefore be interpreted cautiously in terms of practical significance. Furthermore, the total effect was found to be *β* = 0.31 and the indirect effect was *β* = 0.17. The findings are presented in Tables 3, 4 and Figure 1. Due to the cross-sectional design of the study, the findings should be evaluated in terms of indirect statistical relationships between variables rather than causal or temporal inferences. Additionally, the mediation model was re-tested by including gender, age, marital status, daily internet use time, and most frequently used social media platform as covariates. The results remained largely consistent after controlling for these variables. The total indirect effect of FoMO on cyberstalking remained significant (*B* = 0.14, SE = 0.02, 95% CI [0.096, 0.189]). Similarly, the specific indirect effects through abandonment (*B* =  0.055, SE = 0.017, 95% CI [0.024, 0.091]), insufficient self-control (*B* =  0.017, SE = 0.008, 95% CI [0.003, 0.035]), phubbing (*B* =  0.054, SE = 0.018, 95% CI [0.021, 0.092]), abandonment and phubbing (*B* =  0.010, SE = 0.004, 95% CI [0.003, 0.019]), and insufficient self-control and phubbing (*B* =  0.004, SE = 0.002, 95% CI [0.001, 0.007]) were also significant.

**Table 3:** Indirect effects of FoMO on cyberstalking.

Path	*B*	SE	95% Bootstrap CI
Total effect	0.31	0.04	[0.244, 0.383]
Direct effect	0.14	0.04	[0.061, 0.221]
Total indirect effect	0.17	0.03	[0.125, 0.224]
F → A → CS	0.07	0.01	[0.037, 0.117]
F → IS → CS	0.02	0.01	[0.004, 0.051]
F → P → CS	0.06	0.03	[0.036, 0.118]
F → A → P → CS	0.01	0.00	[0.005, 0.025]
F → IS → P → CS	0.01	0.00	[0.003, 0.014]

*B*, unstandardized regression coefficient; SE, standard error. Bootstrap confidence intervals are based on 5,000 resamples. An indirect effect is considered significant when the 95% confidence interval does not include zero. *p < 0.05, **p < 0.01.

**Table 4:** Serial mediation model coefficients.

Predictor	A (*β*, SE, *p*)	IS (*β*, SE, *p*)	P (*β*, SE, *p*)	CS (*β*, SE, *p*)
F	0.18 (0.02), <0.001	0.11 (0.02), <0.001	—	0.14 (0.04), <0.001
A	—	—	—	0.37 (0.09), <0.001
IS	—	—	—	0.19 (0.07), <0.001
P	—	—	—	0.09 (0.02), <0.001
Constant	7.93 (0.35), <0.001	1.82 (0.65), <0.001	1.82 (0.65), <0.001	29.27 (3.55), <0.001

Predictor	A	IS	P	CS
*R^2^*	0.17	0.05	0.33	0.22
*F* (df1, df2)	106.94 (1, 538)	28.60 (1, 538)	88.88 (3, 536)	37.49 (4, 535)
*p*	<0.001	<0.001	<0.001	<0.001

## 5 Discussion and conclusion

### 5.1 Discussion

This study aimed to contribute to the literature by examining several potential predictors of cyberstalking in a sample of adult individuals. Accordingly, a series of mediation models were examined in which FoMO was expected to positively predict abandonment schema, poor self-control schema, and phubbing. The findings revealed that FoMO was significantly associated with cyberstalking both directly and through the mediating variables included in the model. In line with the findings, it may be suggested that online behaviors of individuals in the digital age are not only influenced by technological habits, but also shaped by cognitive and emotional patterns formed in early developmental periods. In addition, the findings indicated that the hypothesized pathways were supported within the tested model.

In the study, it was determined that FoMO was positively related to cyberstalking and that FoMO predicted cyberstalking. Limited studies in the relevant literature are consistent with this finding (Bibi et al., 2023; Gómez-Yepes et al., 2025; Silva Santos et al., 2023). This may be explained by the fact that individuals try to fulfill their need for social bonding and approval through digital platforms. Individuals with high FoMO levels may experience difficulty in controlling their desire to obtain information about the social lives of others (Przybylski et al., 2013), and this may, over time, be associated with cyberstalking behaviors that can be considered as boundary violation and harassment over time.

In the study, path analyses in which early maladaptive schemas were determined as mediating variables in the relationship between FoMO and cyberstalking were examined and it was found that both abandonment schema and insufficient self-control schema played a mediating role in this relationship. When the related literature was examined, no research findings were found in which this model was tested. However, there are studies involving early maladaptive schemas with variables that may be related to cyberstalking. In Arpacı's (2021) study, it was found that mindfulness had a regulatory role in the relationship between early maladaptive schemas and smartphone addiction. In the same study, abandonment, insufficient self-control, social isolation, skepticism, approval seeking and entitlement schemas were positively associated with smartphone addiction. In another study, it was found that inadequate self-control and approval seeking schemas were positively associated with problematic Facebook use (Cudo et al., 2020). In Aras’s (2022) study, a total of 14 schemas, especially abandonment and insufficient self-control, were found to be positively associated with social media addiction. In other studies, early maladaptive schemas were found to be associated with internet addiction (Shajari et al., 2016) and problematic smartphone use (Arpacı, 2023). The findings suggest that abandonment and insufficient self-control schemas may represent theoretically relevant cognitive vulnerabilities associated with the relationship between FoMO and cyberstalking-related behaviors. Individuals may experience fear of rejection or exclusion in social relationships in line with the negative core beliefs they develop at an early age, and these fears may be reflected in increased monitoring behaviors in digital environment. In addition, overuse of online environments and devices may be associated with insufficient self-control schema. From this perspective, it can be said that early maladaptive schemas may be associated with in shaping individual’s behavior in the digital world.

In the study, it is seen that phubbing has a mediating effect in the relationship between FoMO and cyberstalking. When the related literature was examined, no study was found in which cyberstalking and phubbing were addressed together. However, there are many studies showing a positive relationship between FoMO and phubbing to support the current finding (Akeren et al., 2025; Albalá-Genol et al., 2025; Batmaz et al., 2024; González et al., 2025; Tandon et al., 2022; van der Schyff et al., 2022). The mediation of phubbing behavior may point to how digital distraction and social avoidance can feed the tendency to cyberstalking. Phubbing refers to an individual’s avoidance of face-to-face social interactions and focusing their attention on mobile devices (Przybylski et al., 2013). This tendency may increase repetitive and monitoring-oriented observation of one’s social environment online and may be associated with cyberstalking-related behaviors. Moreover, since phubbing may decrease individuals’ social satisfaction, it may also relate to higher FoMO levels.

In another finding of the study, the relationship between FoMO and cyberstalking is mediated by abandonment, insufficient self-control schemas and phubbing together. When the related literature was examined, no research finding was found in which this model was tested. Morkoç (2023) examined the mediating role of well-being in the relationship between early maladaptive schemas and FoMO, nomophobia and phubbing, and found that there was a positive relationship between damaged autonomy and other-orientation schema domains and FoMO and phubbing. The current findings provide a theoretically informed perspective on how individuals’ early cognitive structures and digital behavior patterns may be associated with monitoring-oriented and potentially boundary-crossing online behaviors.

First of all, it was found that abandonment schema and phubbing together mediate the relationship between FoMO and cyberstalking. Abandonment schema is an ingrained belief system that an individual will be abandoned by significant others or left emotionally alone (Young et al., 2003). Individuals with high levels of FoMO may monitor others’ online lives more frequently and obsessively for fear of social exclusion or incompletion (Przybylski et al., 2013). In this context, abandonment schema may intensify sensitivity to social attachment needs and be associated with cyberstalking behavior. In addition, phubbing, that is, shifting one’s attention to the mobile device in social interactions, may reinforce this cycle by reducing social satisfaction (Roberts and David, 2022). Secondly, insufficient self-control schema and phubbing seem to play a similar mediating role together. This schema is characterized by the individual’s difficulty in delaying impulses, emotions, and needs (Young et al., 2003). When combined with FoMO, it may be linked to increased engagement in cyberstalking behavior in search of instant gratification. As a matter of fact, it has been revealed in the literature that low self-control paves the way for uncontrolled and obsessive behaviors in social media use (Wegmann et al., 2017). Similarly, phubbing may contribute to increased time spent in digital environments and reduced face-to-face social interaction (Chotpitayasunondh and Douglas, 2018). These findings suggest that individuals’ digital behaviors may be associated not only with technology use patterns but also with broader psychological and cognitive characteristics. FoMO is not only a social media phenomenon, but also a type of anxiety triggered at cognitive and emotional levels. Early maladaptive schemas such as abandonment and insufficient self-control may be associated with both the severity of this anxiety and the individual’s reactions to this anxiety (Muris, 2006). Therefore, problematic digital behaviors such as cyberstalking may be linked to broader psychological needs and coping mechanisms.

This study aimed to examine the mediating roles of early maladaptive schemas and phubbing behavior in the relationship between FoMO and cyberstalking behaviors. The findings indicated that both early maladaptive schemas and phubbing behavior played a significant mediating role in this relationship. These results suggest that individuals with high FoMO levels may show an increased tendency to cyberstalking, especially when maladaptive cognitive patterns and digital social avoidance behaviors are present. The findings highlight the importance of considering cognitive vulnerabilities and digital habits in understanding problematic online behaviors. In particular, it is thought that early maladaptive schemas such as abandonment and insufficient self-control may increase the emotional reactivity associated with FoMO and push individuals to compulsively monitor others’ online activities. Likewise, phubbing behavior may not only reflect these patterns, but also play a reinforcing role.

### 5.2 Limitations and future research

It can be thought that the study will contribute to the literature in order to determine the dynamics underlying cyberstalking behavior, which has become widespread in recent years and has many negative effects. However, the study has some limitations. In the current study, only adult individuals in a certain age range were included. Future research should include participants with a wider range of ages, life experiences and cultural backgrounds. It may be recommended to conduct future studies especially with adolescents who use the internet and social media intensively. This research is a quantitative study conducted with the survey method. In this context, it may be recommended to conduct qualitative study to learn the views and perspectives of the participants about the concepts, longitudinal study to see the change and development of the concepts and findings over time, and experimental study to examine the effect of concepts on each other. Testing the links between cyberstalking and other variables in different cultural contexts may improve understanding of digital behaviors. In this study, cyberstalking and the variables of FoMO, phubbing and early maladaptive schemas were examined. In future research, it may be recommended to examine different variables that may be related to cyberstalking. In this study, only abandonment and insufficient self-control schemas were included among early maladaptive schemas. In order to better understand the nature of cyberstalking, studies with other early maladaptive schemas can be conducted. In addition, data were collected using self-report measurement tools. Since this may be a limitation, generalizations should be made with caution and results should be evaluated accordingly. Furthermore, because all variables were measured using self-report instruments collected at a single time point, the findings may also be influenced by common method variance, social desirability tendencies, memory-related biases, and temporal ambiguity among the study variables. Finally, collecting the data through an online form can also be considered as a limitation. In addition, the use of inclusion criteria such as “using the internet every day” and “having at least one social media account” in the study may have led to the sample consisting of more online-active individuals compared to the general adult population. Therefore, FoMO, phubbing, and cyberstalking levels may have been observed to be higher compared to the general adult population. Accordingly, the findings should be interpreted within the context of a relatively more digitally active adult sample, and caution is warranted when generalizing the results to broader populations with different levels of internet and social media engagement. This situation may cause the findings to be more explanatory for adults who engage in more intensive online interaction and may limit the generalizability of the results. In addition, although the proposed model explained a statistically meaningful proportion of variance in cyberstalking, a substantial portion of the variance remained unexplained, indicating that additional psychological, interpersonal, and contextual variables may also contribute to cyberstalking behaviors. Furthermore, measurement invariance across demographic variables such as sex and age was not examined in the present study and should be addressed in future research. Therefore, it is recommended that the model be retested in subsequent studies with more heterogeneous samples in terms of internet and social media usage intensity.

Psychological intervention programs can potentially focus on helping individuals recognize early maladaptive schemas such as abandonment and insufficient self-control, and develop coping skills to address these schemas. However, the cross-sectional design of the study and findings based on indirect effects do not provide direct evidence for establishing a causal relationship between these variables or that a specific intervention will reduce cyberstalking behavior. Accordingly, the proposed serial mediation model should be interpreted as a theoretically informed associational framework rather than evidence of temporal or causal ordering among the variables. The sequential ordering represented in the serial mediation model therefore reflects a theoretically derived analytical structure rather than empirically established temporal sequencing among the variables. Similarly, psychological interpretations discussed in the manuscript should be considered theoretical inferences derived from the observed associations rather than direct empirical evidence of underlying psychological mechanisms. However, when indirect effect findings are evaluated together, it is seen that indirect pathways related to the abandonment schema appeared relatively more consistent and pronounced compared to the other indirect pathways examined in the study; whereas the indirect effect related to the insufficient self-control schema provides relatively more limited evidence, with a smaller magnitude and a confidence interval lower bound close to zero (Table 3). Therefore, practical implications related to the abandonment schema may be interpreted with relatively greater consistency, whereas findings related to the insufficient self-control schema should be evaluated more cautiously. In addition, the relatively small number of items in these schema subdimensions may have contributed to comparatively lower internal consistency coefficients in the present sample, as shorter subscales may yield relatively lower Cronbach’s alpha values in social science research (Taber, 2018). Cognitive-behavioral approaches, particularly schema therapy, may provide a useful theoretical framework for understanding how maladaptive cognitive patterns could be associated with problematic digital behaviors (Rafaeli et al., 2011; Young et al., 2003). FoMO-focused approaches discussed in the literature may consider not only technology use patterns but also broader psychological characteristics such as attachment-related concerns and self-regulation difficulties. Mindfulness-based practices, in particular, have been shown to reduce FoMO levels and problematic social media use (Brown and Ryan, 2003; Elhai et al., 2021). In this context, considering that FoMO and related digital behavior patterns (e.g., phubbing) may be associated with cyberstalking, awareness-based approaches may be considered as potential intervention targets that could be explored in future research and practice contexts. Phubbing behavior has been associated with reduced closeness and empathy in interpersonal relationships in previous studies. Therefore, awareness-raising practices related to digital etiquette and healthy online communication skills may be considered in future research and practice contexts (Chotpitayasunondh and Douglas, 2018; Roberts and David, 2022). Schools, universities, and mental health professionals may benefit from considering the broader psychological characteristics associated with digital behaviors rather than evaluating these behaviors solely within the context of addiction. However, longitudinal and intervention-based (experimental) studies need to be conducted in the future to evaluate the effectiveness of such intervention approaches in reducing cyberstalking behavior.

### 5.3 Conclusion

These results suggest that not only technological factors but also developmental and cognitive components should be considered in understanding individuals’ digital behaviors. The findings may suggest that broader psychological and cognitive characteristics could also be considered when discussing FoMO-related digital behaviors in adults, rather than focusing exclusively on technology use patterns. This study tested a new model to explain how early maladaptive schemas and phubbing may mediate the relationship between FoMO and cyberstalking. The study addressed a gap regarding negative digital behaviors such as cyberstalking, FoMO, and phubbing in Turkish adults. Moreover, the fact that the study combines cognitive and behavioral dimensions in the digital context may provide a theoretical basis for future studies examining mindfulness-based and cognitively oriented approaches to digital behavior patterns as well as interventions targeting schema change. It is thought that the study will contribute to the literature in order to use social media and digital devices more effectively and positively.

## Data Availability

The raw data supporting the conclusions of this article will be made available by the authors, without undue reservation.
